# Secreted proteins MDK, WFDC2, and CXCL14 as candidate biomarkers for early diagnosis of lung adenocarcinoma

**DOI:** 10.1186/s12885-023-10523-z

**Published:** 2023-01-31

**Authors:** Junfeng Li, Jianjie Li, Huifeng Hao, Fangliang Lu, Jia Wang, Menglei Ma, Bo Jia, Minglei Zhuo, Jingjing Wang, Yujia Chi, Xiaoyu Zhai, Yuyan Wang, Meina Wu, Tongtong An, Jun Zhao, Fan Yang, Ziping Wang

**Affiliations:** 1 Departments of Thoracic Medical Oncology, Key Laboratory of Carcinogenesis and Translational Research, Peking University Cancer Hospital & Institute, Beijing, 100142 China; 2 Department of Integration of Chinese and Western Medicine, Key Laboratory of Carcinogenesis and Translational Research (Ministry of Education, Beijing), Peking University Cancer Hospital & Institute, Beijing, 100142 China; 3 Department of Thoracic Surgery II, Key Laboratory of Carcinogenesis and Translational Research (Ministry of Education, Beijing), Peking University Cancer Hospital & Institute, Beijing, 100142 China; 4 Department of Thoracic Surgery, Peking University People’s Hospital, Beijing, 100044 China

**Keywords:** Lung adenocarcinoma (LUAD), Liquid biopsy, Early diagnosis, Biomarker

## Abstract

**Background:**

Early diagnosis of lung adenocarcinoma (LUAD), one of the most common types of lung cancer, is very important to improve the prognosis of patients. The current methods can’t meet the requirements of early diagnosis. There is a pressing need to identify novel diagnostic biomarkers. Secretory proteins are the richest source for biomarker research. This study aimed to identify candidate secretory protein biomarkers for early diagnosis of LUAD by integrated bioinformatics analysis and clinical validation.

**Methods:**

Differentially expressed genes (DEGs) of GSE31210, gene expression data of early stage of LUAD, were analyzed by GEO2R. Upregulated DEGs predicted to encode secreted proteins were obtained by taking the intersection of the DEGs list with the list of genes encoding secreted proteins predicted by the majority decision-based method (MDSEC). The expressions of the identified secreted proteins in the lung tissues of early-stage LUAD patients were further compared with the healthy control group in mRNA and protein levels by using the UALCAN database (TCGA and CPTAC). The selected proteins expressed in plasma were further validated by using Luminex technology. The diagnostic value of the screened proteins was evaluated by receiver operating characteristic (ROC) analysis. Cell counting kit-8 assay was carried out to investigate the proliferative effects of these screened proteins.

**Results:**

A total of 2183 DEGs, including 1240 downregulated genes and 943 upregulated genes, were identified in the GSE31210. Of the upregulated genes, 199 genes were predicted to encode secreted proteins. After analysis using the UALCAN database, 16 molecules were selected for further clinical validation. Plasma concentrations of three proteins, Midkine (MDK), WAP four-disulfide core domain 2 (WFDC2), and C-X-C motif chemokine ligand 14 (CXCL14), were significantly higher in LUAD patients than in healthy donors. The area under the curve values was 0.944, 0.881, and 0.809 for MDK, WFDC2, and CXCL14, 0.962 when combined them. Overexpression of the three proteins enhanced the proliferation activity of A549 cells.

**Conclusions:**

MDK, WFDC2, and CXCL14 were identified as candidate diagnostic biomarkers for early-stage LUAD and might also play vital roles in tumorigenesis.

**Supplementary Information:**

The online version contains supplementary material available at 10.1186/s12885-023-10523-z.

## Introduction

According to the GLOBOCAN 2020 estimates, lung cancer is the second most frequent cancer (11.4%) and remains the first leading cause of cancer deaths (18%) [1]. Lung adenocarcinoma (LUAD) is the most common pathological subtype (40%) that has a high risk of developing micro-metastases in the early stage [2]. The 5-year relative survival rate drops dramatically from 85% for stage IA to 6% for stage IV [3]. About 70% of LUAD patients were at stage III ~ IV at diagnosis, which implies that less than 20% of the patients are expected to survive for 5 years [4]. Therefore, it is urgent to establish an effective screening and diagnosis system for early-stage LUAD.

Low-dose spiral computed tomography is currently widely used for early screening of lung cancer. As one of the effective approaches for early detection of lung cancer in high-risk populations, it has been reported to reduce the mortality of lung cancer by 20% [5, 6]. However, the high rate of misdiagnosis and missed diagnosis is a major problem [7], that leads to unnecessary invasive tests or surgery. Liquid biopsy (e.g., blood) is a convenient and minimally-invasive method that can detect abnormally expressed proteins or other molecules in body fluids. Circulating nucleic acids, circulating tumor cells, and non-coding RNAs (lncRNA and miRNA), as well as exosomes, are new types of biomarkers for liquid biopsy to aid in the diagnosis of tumors [8]. Increasing evidence showed the diagnostic value of non-coding RNAs [9–11], DNA methylation [12], and protein [13] in LUAD.

Proteins, carrying out various functions inside and outside of the cell, are the vital functional executors in the life processes. Protein biomarkers are particularly popular with the availability of a wide range of analytical instrumentation. Secretory proteins, encoded by secreted genes, are transported out of the cell to extracellular fluid, such as blood plasma, under the guidance of a signal peptide. Secretory proteins are the richest source for biomarker research [6, 14]. Identification of differentially expressed secretory proteins from blood or other body fluids has become an effective method for biomarker research. The combination of several protein biomarkers, such as CEA, CA125, CA153, and Cyfra21–1, can significantly improve the diagnosis accuracy of lung cancer [15]. However, the research on screening and identification of clinical biomarkers for early diagnosis of LUAD is still lacking. There are no highly sensitive and specific protein biomarkers used in clinical practice up to now.

To screen more sensitive and specific secretory protein biomarkers for diagnosis of early-stage LUAD, we used an integrated transcriptomic and computational analysis in combination with clinical validation. The gene expression profiles of 114 stage IA LUAD patients and 20 healthy donors were obtained from the Gene Expression Omnibus (GEO) database to identify differentially expressed genes (DEGs). Upregulated proteins are easier to detect in biofluids. Therefore, the upregulated secretory proteins were predicted by taking the interaction of upregulated genes and secretory genes list of the human protein atlas (HPA) database. The target proteins were then filtered by taking the interaction with the list of detectable proteins on the Luminex platform. The mRNA and protein level expressions of the identified secreted proteins were verified in the UALCAN database (The Cancer Genome Atlas [TCGA] and Clinical Proteomic Tumor Analysis Consortium [CPTAC], respectively). Plasma samples of stage I LUAD patients and healthy control were collected for further validation of the identified secretory proteins by using the Luminex assay.

## Methods

### Study workflow for identification of candidate biomarkers

**Fig. 1:**
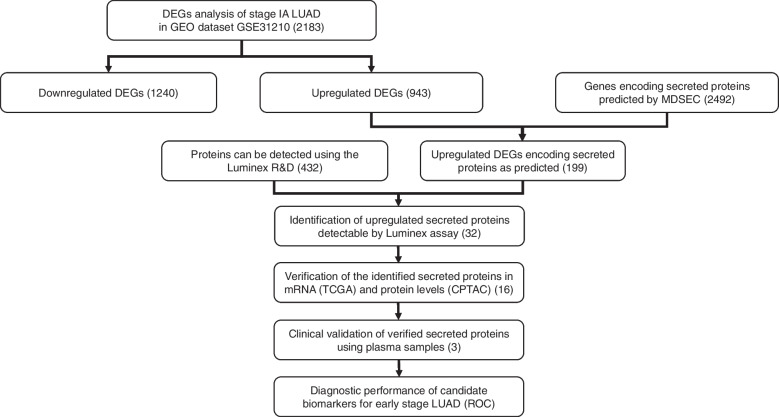
The workflow for identification of candidate biomarkers for early diagnosis of lung adenocarcinoma (LUAD). The workflow contains two parts: bioinformatics analysis and clinical validation. The bioinformatics analysis contains upregulated differentially expressed genes (DEGs) screening and genes encoding secreted proteins predicted by the majority decision-based method. The expressions of the identified secreted proteins are validated using the TCGA and CPTAC databases. The clinical validation was carried out using the plasma samples of 26 early-stage LUAD patients and 11 healthy donors

An overall workflow for the identification of candidate diagnostic biomarkers for early-stage LUAD is illustrated in Fig. 1. Briefly, upregulated secreted proteins were screened based on the following criteria: (i) corresponding genes were upregulated in the early-stage LUAD from the GEO dataset (GSE31210); (ii) corresponding upregulated genes encode secreted proteins as predicted by the majority decision-based method (MDSEC); and (iii) secreted proteins available on the Luminex Assay Customization menu. Expressions of the identified secreted proteins were validated in the mRNA (TCGA) and protein (CPTAC) levels using the UALCAN database. The proteins identified were then verified using plasma samples from 26 stage I LUAD patients and 11 healthy donors. The diagnostic performance of the candidate secreted proteins was analyzed by receiver operator characteristic (ROC) curve analysis. This study was approved by the Medical Ethics Committee of the Peking University Cancer Hospital (No. 2021KT104). The study was conducted in accordance with the Declaration of Helsinki (as revised in 2013). Written informed consent was obtained from all participants.

### Data acquisition and differentially expressed genes (DEGs) analysis

The early LUAD gene expression profile data and corresponding clinical data were downloaded from the GEO database. The dataset GSE31210, including 114 stage IA LUAD and 20 normal control, was used to screen DEGs by using an online tool GEO2R. The criteria were set as *P* < 0.05 and |log2-fold change| ≥ 1. A volcano map of DEGs was drawn by using the ggplot2 package of the R software. A heat map of the 32 selected DEGs was generated by Morpheus, an online tool of Broad institute.

### Identification of upregulated DEGs encoding secreted proteins

Upregulated DEGs encoding secreted proteins were screened by taking the intersection of the upregulated DEGs with the gene list encoding secreted proteins predicted by using a majority decision-based method (MDSEC), a multi-decision-making algorithm based on the SignalP4.0, Phobius, and SPOCTOPU, in the HPA database. The predicted secreted proteins were further filtered using the data of validated secreted proteins from the Luminex platform. The preliminary screening results were displayed on the Venn diagram using the web tool (bioinformatics.psb.ugent.be/webtools/Venn/).

### Gene ontology (GO) and Kyoto encyclopedia of genes and genomes (KEGG) analysis

The Gene Ontology (GO) and Kyoto Encyclopedia of Genes and Genomes (KEGG) analysis was performed in the Database for Annotation, Visualization and Integrated Discovery (DAVID) [16]. The upregulated DEGs encoding secreted proteins were submitted to the website of DAVID. The GO enrichment analysis includes biological processes (BP), cellular components (CC), and molecular function (MF) annotations for biological processes. The annotations of the genes with biological processes and KEGG pathway enrichment were carried out by the website https://www.bioinformatics.com.cn, a free online platform for data analysis and visualization.

### Verification of the identified secreted proteins in the UALCAN database

The UALCAN is an interactive web resource that provides mRNA analysis of the TCGA database and protein analysis of the CPTAC database. The secreted proteins identified and their corresponding encoding genes were submitted to the UALCAN for verification in more cases of LUAD. The mRNA-level verification was performed on the TCGA database (http://ualcan.path.uab.edu/analysis.html), including 515 LUAD patients (277 stage I, 125 stage II, 85 stage III, and 28 stage IV) and 59 normal controls. The protein-level verification was performed on the CPTAC database (http://ualcan.path.uab.edu/analysis-prot.html), including 111 LUAD patients (59 stage I, 30 stage II, 21 stage III, and 1 stage IV) and 11 normal controls.

### Plasma sample collection and preparation

Plasma samples of 26 stage I LUAD patients and 11 healthy donors were collected from the Department of Thoracic Surgery II, Peking University Cancer Hospital. The baseline demographic characteristics of the patients and healthy controls were generally comparable (Table 1). Freshly collected blood samples were temporarily stored at 4 °C for plasma separation within 4 hours. The plasma samples were obtained by centrifugation at 2000 g for 10 minutes at 4 °C and stored in a − 80 °C refrigerator for analysis.

**Table 1:** Characteristics

Characteristics	All	Health donors	Stage I LUAD
	(***n*** = 37)	(***n*** = 11)	(***n*** = 26)
**Age, years**			
Median [range]	57 [37–78]	56 [41–72]	57 [37–78]
**Sex,** ***n*** **(%)**			
Female	21 (56.7)	5 (45.5)	16 (61.5)
Male	16 (43.3)	6 (54.5)	10 (38.5)
**Smoking,** ***n*** **(%)**			
Yes	19 (51.4)	3 (27.3)	16 (61.5)
No	18 (48.6)	8 (72.7)	10 (38.5)

**Note:** *LUAD* Lung adenocarcinoma

### Plasma validation of the verified secreted proteins by Luminex assay

Plasma levels of the verified secreted proteins were validated by using the customized magnetic bead-based multiplex assay (Luminex R&D Systems, USA). All plasma samples were diluted to 25-fold and plated in duplicate. The protein concentrations were measured according to the manufacturer’s instructions. The plates were read using the Luminex X-200™ instrument (Thermo Fisher Scientific, Waltham, MA, USA). Data were preprocessed and analyzed with the Luminex xPONENT software.

### Receiver operating characteristic (ROC) analysis

The diagnostic performance of each candidate biomarker or their combination was assessed by the ROC analysis. The area under the ROC curve (AUC) was calculated using the MedCalc statistical software. The sensitivity, specificity, positive predictive value (PPV), negative predictive value (NPV), and diagnostics odds ratio (DOR) of the 16 proteins in the diagnosis of early-stage LUAD were calculated using the diagnostic test evaluation calculator of MedCalc statistical software.

### Plasmids

Full-length gene sequences encoding MDK, WFDC2, and CXCL14 were obtained and cloned together with 6 × His tag into the lentiviral vector pCDH-CMV-MCS-EF1-Puro (CD510B-1, System Biosciences). Three packaging plasmids pPACKH1-GAG, pPACKH1-REV, and pVSV-G were obtained from the System Biosciences.

### Lentivirus transduction into A549 cells

Human embryonic kidney HEK293T cells and LUAD A549 cells (Eallbio, Beijing, China) were cultured in 1640 medium with 10% fetal bovine serum, 2 mmol/L L-glutamine, 100 U/mL penicillin, and 100 mg/mL streptomycin. HEK293T cells were co-transfected with each of the lentiviral vectors expressing MDK, WFDC2, and CXCL14 or empty vector with packaging plasmids pPACKH1-GAG, pPACKH1-REV, and pVSV-G in a 3:1:1:1 ratio using the TurboFect™ Transfection Reagent (Thermo Scientific) following the manufacturer’s protocol. Viral supernatants were collected 72 h after transfection. A549 cells were divided into four groups and infected with each of three protein expression lentiviruses or empty lentiviruses, respectively. The cells in each group were then treated with puromycin for 3 weeks for the selection of stable transfectants. The selected cells were cultured in 6 well plates for 48 h and harvested for expression analysis by western blot.

### Western blot analysis

The harvested cells were lysed in the RIPA lysis buffer. The targeted proteins (MDK, WFDC2, and CXCL14) were separated in 12% SDS-PAGE gel and then transferred to PVDF (Millipore) membranes. After being blocked with 5% milk, the membranes were probed with 6 × His tag antibody and GAPDH antibody (Solarbio life science, China), followed by washing and incubation with HRP-conjugated secondary antibody. Reactive bands were detected by enhanced chemiluminescence (Millipore reagents).

### Cell counting kit-8 (CCK-8) assay

The proliferative capacity of the A549 cells in each group was tested by CCK-8 assay. The cells were trypsinized and seeded into 96-well plates at a density of 4 × 10^3^ cells/well in a volume of 100 μl. At the 2nd and 48th hours, the cells were incubated with 10 μl CCK-8 reagent solution (Dojindo Molecular Technologies, Inc. Japan) for 3 hours under regular culture conditions. The optical density was determined at 450 nm. Each experiment was repeated five times.

### Survival analysis

The Kaplan-Meier plotter tool (http://kmplot.com/analysis/) was used to analyze the correlation between the expression of candidate biomarkers and overall survival in 672 LUAD patients. Patients were divided into two groups according to the median expression levels of candidate biomarkers and the Kaplan-Meier survival curve was then plotted.

### Statistical analysis

Quantitative values were expressed as mean ± standard error of the mean (SEM). A comparison of protein expression levels was made by Student’s t-test using the GraphPad Prism version 9.0.0 software. *P* value < 0.05 was considered statistically significant.

## Results

### Upregulated DEGs encoding detectable secreted proteins

**Fig. 2:**
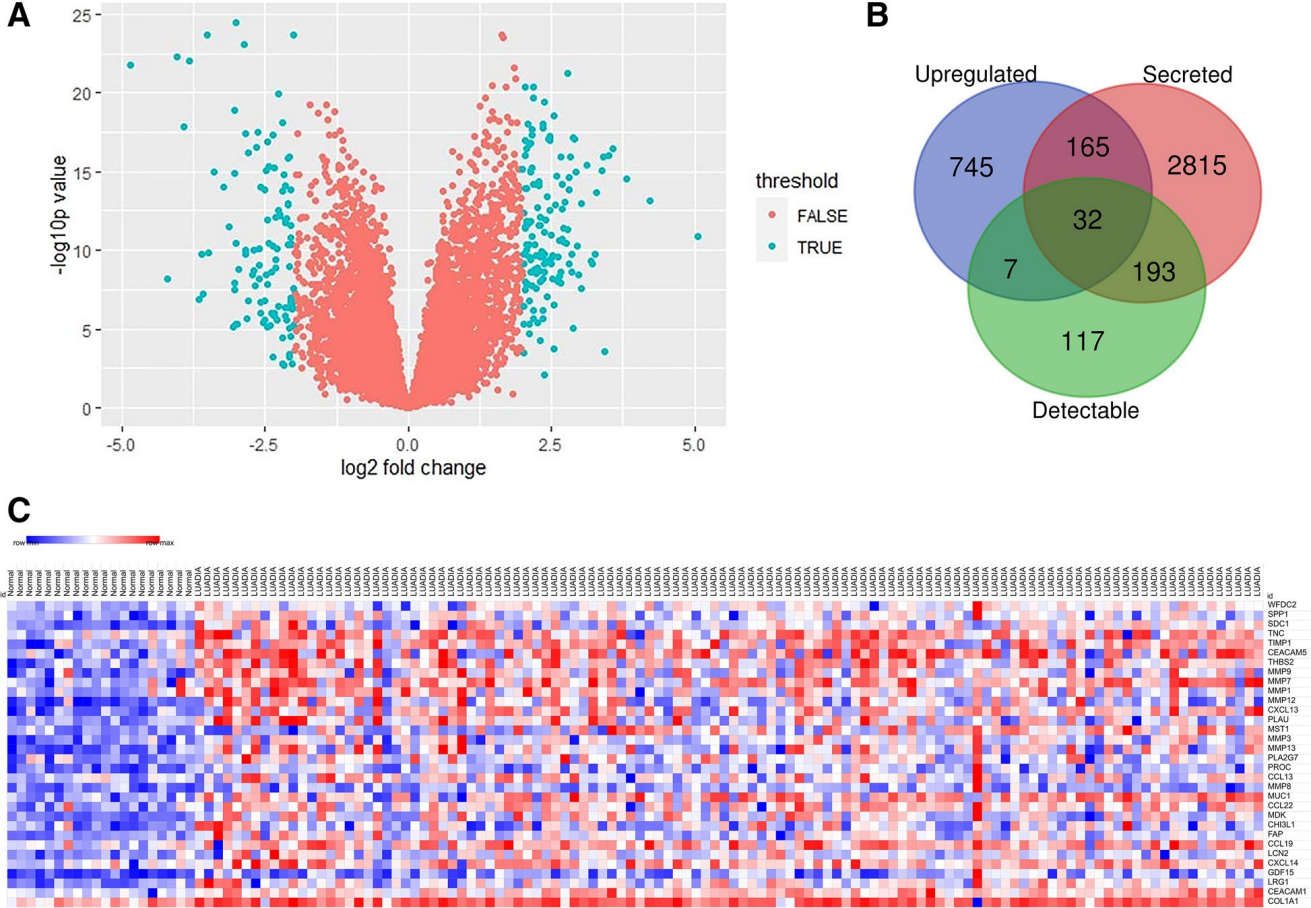
Identification of differentially expressed genes (DEGs) encoding secreted proteins using the GEO dataset GSE31210. (**A**) Volcano map exhibits DEGs in lung tissues of 20 normal control and 114stage IA LUAD patients in the GEO dataset GSE31210: Green plots represent the DEGs (fold change> 2; a *p*-value < 0.05); red plots represent genes not differentially expressed. (**B**) Venn diagram of upregulated DEGs in the stage IA LUAD, genes encoding secreted proteins and genes encoding proteins detectable using the Luminex assays (R&D). (**C**) Heatmap of the expression of 32 upregulated DEGs identified as shown in the Venn diagram

To identify DEGs in early-stage LUAD, GEO dataset GSE31210, including 114 stage IA LUAD patients and 20 normal control, was downloaded for analysis. There were 2183 DEGs in the early stage LUAD, including 1240 downregulated DEGs and 943 upregulated DEGs (Fig. 2 A). Of the 943 upregulated DEGs, 199 were predicted to encode secretory proteins after taking interaction with the genes encoding secretory proteins predicted by MDSEC.

**Fig. 3:**
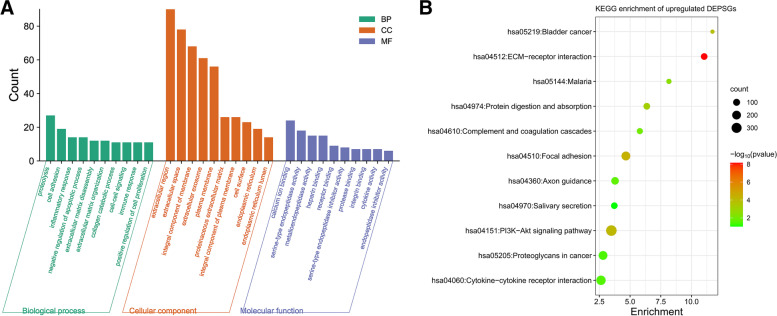
Functional enrichment analysis of 199 upregulated differentially expressed genes (DEGs) encoding secreted proteins in early-stage LUAD. (**A**) GO functional classification of 199 upregulated DEGs (Biological Process (BP): biological process, MF: Molecular Function, CC: Cellular Component) annotations; (**B**) Kyoto Encyclopedia of Genes and Genomes (KEGG) pathway analysis of the most significantly enriched terms for the upregulated DEGs

The DAVID was used to output GO annotation and KEGG pathway enrichment of 199 upregulated DEGs predicted to encode secreted proteins. As shown in Fig. 3, the calcium-iron binding of molecular function, the biology progress of proteolysis, and the ECM-receptor interaction were the most significantly enriched pathways.

Target proteins were further screened by taking the intersection of the 199 secreted proteins encoded by upregulated DEGs as predicted and secreted proteins that can be detected by Luminex assay according to the product catalog (R&D Systems). As shown in Fig. 2 B, a total of 32 secreted proteins encoded by upregulated DEGs and detectable on the Luminex platform were obtained. The 32 gene expression data in GSE31210 were visualized in the heatmap (Fig. 2 C).

### Verification of the secreted proteins identified in the UALCAN database

All the 32 secreted proteins identified were submitted to the UALCAN to verify their relative expressions in LUAD. Sixteen proteins were expressed much higher in early-stage LUAD than in healthy control in both the mRNA (Supplementary Fig. 1) and protein levels (Supplementary Fig. 2), including Midkine (MDK), Secreted Phosphoprotein 1 (SPP1), TIMP Metallopeptidase Inhibitor 1 (TIMP1), Thymidine Kinase 1 (TK1), Tenascin C (TNC), Thrombospondin 2 (THBS2), Collagen Type I Alpha 1 Chain (COL1A1), Matrix Metallopeptidase 12 (MMP12), Plasminogen Activator, Urokinase (PLAU), C-X-C Motif Chemokine Ligand 13 (CXCL13), Fibroblast Activation Protein Alpha (FAP), Syndecan 1 (SDC1), C-X-C Motif Chemokine Ligand 14 (CXCL14), CEA Cell Adhesion Molecule 1 (CEACAM1), WAP Four-Disulfide Core Domain 2 (WFDC2) and Mucin 1, Cell Surface Associated (MUC1). Therefore, these 16 proteins were selected for further validation.

### Higher plasma levels of MDK, WFDC2, and CXCL14 in patients with early-stage LUAD

**Fig. 4:**
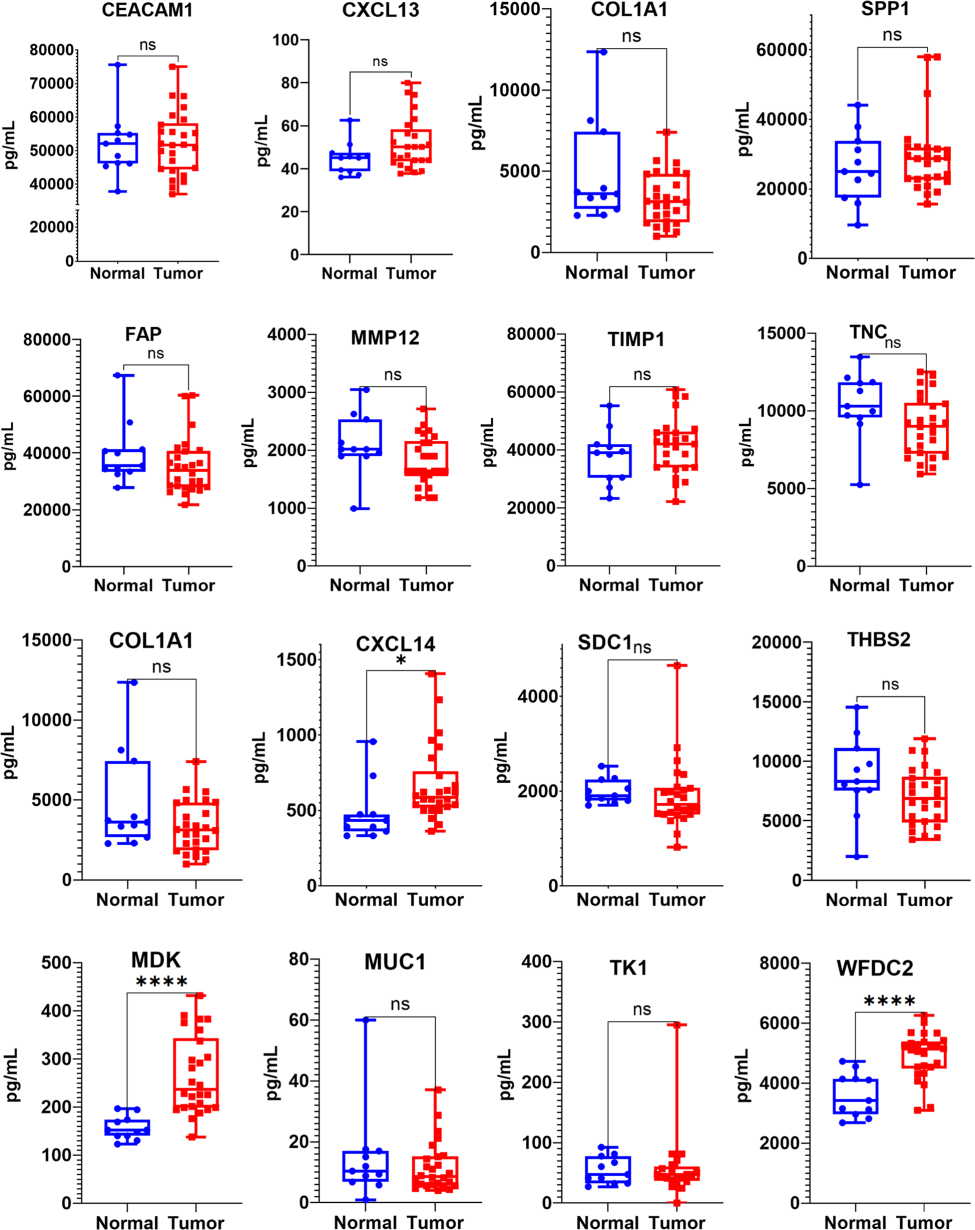
Upregulated plasma MDK, WFDC2, and CXCL14 levels in stage I LUAD patients. Plasma levels of 16 proteins in 26 stage I LUAD and 11 healthy donors were tested by Luminex assay. Each sample repeats twice. The MDK, WFDC2, and CXCL14 were upregulated in the plasma of stage I LUAD patients (*p* < 0.05)

To further validate plasma levels of the 16 proteins, Luminex technology was used to evaluate the protein concentration in plasma samples from 26 stage I LUAD patients and 11 healthy donors. Plasma levels of MDK, WFDC2, and CXCL14 were significantly higher in the LUAD patients than in the health donors (Fig. 4).

### Diagnostic performance of MDK, WFDC2, and CXCL14

**Fig. 5:**
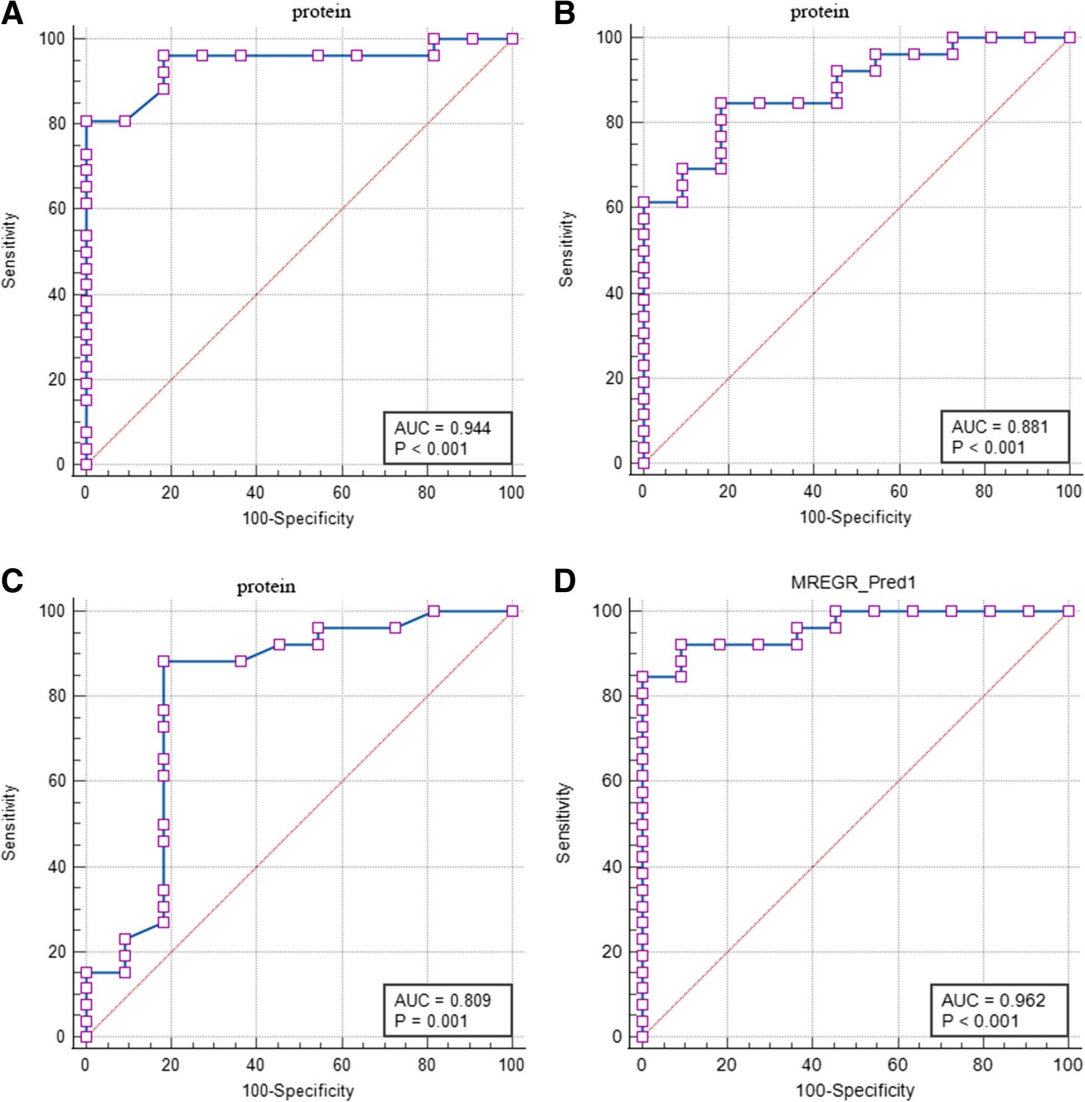
Diagnostic performance of MDK, WFDC2, CXCL14 or their combination assessed by receiver operation characteristic (ROC) curve analysis. ROC curves of MDK (**A**) WFDC2 (**B**) CXCL14 (**C**) or their combination (**D**)

To evaluate the diagnostic performance of three secreted proteins (MDK, WFDC2, and CXCL14) for early-stage LUAD, the ROC analysis was performed on all 16 proteins that were demonstrated to be highly expressed in patients with early-stage LUAD in the UALCAN database. As shown in Table 2 MDK, WFDC2 and CXCL14 scored higher than the other proteins, with respect to sensitivity specificity PPV, NPV, and DOR. The AUC was 0.944, 0.881, and 0.809, respectively (Fig. 5 A~C). The combination of MDK, WFDC2, and CXCL14 resulted in an AUC of up to 0.962 (Fig. 5 D).

**Table 2:** Sensitivity, specificity, PPV, NPV, and DOR values of 16 proteins in early-stage LUAD

	Sensitivity (%)	Specificity (%)	PPV (%)	NPV (%)	DOR
MDK	80.77	100.00	100.00	68.75	∞
WFDC2	84.62	81.82	91.67	69.23	24.75
CXCL14	88.46	81.82	92.00	75.00	34.50
CXCL13	50.00	81.82	86.67	40.91	4.50
CEACAM1	69.23	9.09	64.29	11.11	0.23
COL1A1	30.77	100.00	100.00	37.93	∞
FAP	46.15	90.91	92.31	41.67	8.57
SDC1	50.00	100.00	100.00	45.83	∞
MMP12	53.85	90.91	93.33	45.45	11.67
TNC	69.23	81.82	90.00	52.94	10.13
THBS2	57.69	81.82	88.24	45.00	6.14
SPP1	96.15	27.27	75.76	75.00	9.38
MUC1	50.00	72.73	81.25	38.10	2.67
TK1	26.92	100.00	100.00	36.67	∞
PLAU	65.38	54.55	77.27	40.00	2.27
TIMP1	53.85	81.82	87.50	42.86	5.25

Abbreviation: *PPV* positive predictive value, *NPV* negative predictive value, *PPV* positive predictive value, *NPV* negative predictive value, *DOR* Diagnostic Odds Ratio

### Ectopic expression of MDK, WFDC2, or CXCL14 enhanced proliferation of A549 cells

**Fig. 6:**
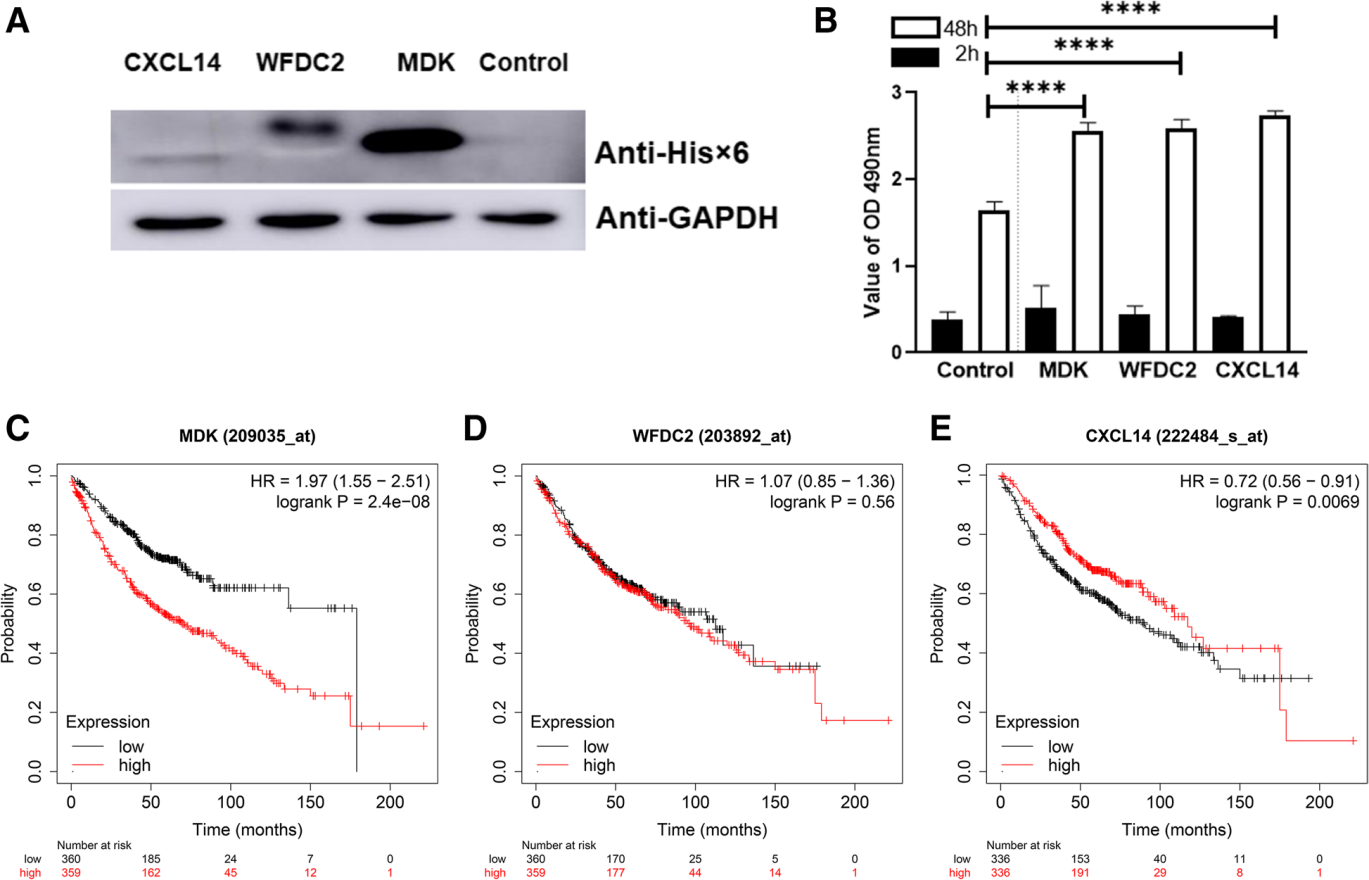
Ectopic expression of MDK, WFDC2, or CXCL14 enhanced the proliferative activity of lung cancer cells. (**A**) Expression of MDK, WFDC2, and CXCL14 in A549 cells infected with recombinant lentivirus expressing 6xHis tagged MDK, WFDC2, or CXCL14 was determined by Western blot analysis with anti-6xHis monoclonal antibody. (**B**) The effect of MDK, WFDC2, or CXCL14 expression on the proliferative activity of A549 cells was assessed by CCK8 assay. Cells were infected with the corresponding lentivirus and screened by puromycin. Assays were performed at least three times. Data are expressed mean ± SEM. Kaplan-Meier survival analysis of the prognostic significance of MDK (**C**) WFDC2 (**D**) or CXCL14 (**E**) expression in LUAD patients using the Kaplan-Meier plotter database

To study the effect of ectopic expressions of three proteins on the proliferative activity of lung cancer cells, recombinant lentiviruses carrying MDK, WFDC2, or CXCL14 were constructed and transduced into A549 cells, respectively. Western blot analysis showed higher expressions of MDK, WFDC2, and CXCL14 three weeks after transduction (Fig. 6 A, Supplementary Fig. 3). Ectopic expression of MDK, WFDC2, and CXCL14 enhanced the proliferative activity of the A549 cells when compared with blank control (Fig. 6 B). Furthermore, high MDK expression was correlated with worse overall survival (Fig. 6 C) (*P* < 0.001), while high WFDC2 expression was not correlated with overall survival (Fig. 6 D). High expression of CXCL14 was correlated with better overall survival (*P* < 0.01, Fig. 6 E).

## Discussion

To identify candidate biomarkers for diagnosis of early-stage LUAD, we analyzed the upregulated secretory proteins that are easier to detect in biofluids in early-stage LUAD using the GEO and TCGA database and validated the expression of these proteins in the plasma samples of early-stage LUAD patients. The results showed that MDK, WFDC2, and CXCL14 were upregulated in the plasma samples of early-stage LUAD patients and might act as the predictive biomarkers for early-stage diagnosis. They might also play vital roles in the tumorigenesis of LUAD.

Many protein biomarkers, such as serum amyloid A, Hp, and complement components 9 (C9) and 4d (C4d), have been identified and validated in serum or plasma samples of lung cancer [17]. In the present study, the results of ROC analysis showed that MDK, WFDC2, and CXCL14 could serve as candidate biomarkers for early diagnosis of LUAD. Meanwhile, combing them together resulted in more effective diagnostic performance.

Of the three screened proteins, MDK encodes a member of a small family of secreted growth factors that bind to heparin and respond to retinoic acid. It functions as a cytokine and growth factor with complex biological functions, including cell proliferation and differentiation, tissue repair, inflammatory response, angiogenesis, and tumorigenesis [18], contributing to both normal tissue homeostasis and disease development. As a soluble secreted protein, MDK is abnormally expressed at high levels in various human malignancies [19] including lung cancer. MDK expression has been determined in blood, urinary, and tumor tissues of cancer patients. It could serve as a valuable diagnostic or prognostic biomarker for non-small cell lung cancer [20]. Also, MDK was found to be highly expressed in stage IA LUAD patients by using single-cell RNA sequencing [21]. Partially consistent with the above-mentioned results, our study showed the upregulated MDK in the plasma of LUAD patients, suggesting MDK as a candidate biomarker for early-stage LUAD. Survival analysis showed that higher expression of MDK was associated with poor overall survival of LUAD patients (Fig. 6 C). All these results indicate that MDK might serve as an early diagnostic and prognostic biomarker for LUAD.

WFDC2, a glycoprotein belonging to the family of whey acidic four-disulfide core proteins, might play a role in sperm maturation. It has been recently characterized as a sensitive ovarian cancer biomarker [22], approved by the Food Drug Administration. WFDC2 was found to be expressed in multiple tissues in the respiratory tracts, salivary gland, and oral cavity, as well as in renal tubular epithelial cells [23]. It plays important roles in the development of lung tissue. Homozygous WFDC2 deletion mice showed increased apoptosis of type-I alveolar cells in lung tissues [24] and suffered severe dyspnea. WFDC2 protein is elevated in malignant pleural effusion and has diagnostic value in LUAD, squamous cell carcinoma, and small cell carcinoma [25]. Our results showed elevated levels of WFDC2 in the plasma of early-stage LUAD patients and enhanced proliferation of lung cancer cells with ectopic expression of WFDC2. Therefore, WFDC2 might play dual roles: i) maintaining growth and development in the organogenesis of the lung; and ii) inducing tumorigenesis and development.

CXCL14, a member of the CXC subfamily of chemokines, mainly induces the chemotaxis of monocytes, dendritic cells, and macrophages. CXCL14 is upregulated in glioblastoma, osteosarcoma, ovarian cancer, thyroid carcinoma, and endometrioid carcinoma, while downregulated in melanoma, colorectal cancer, and hepatocellular carcinoma [26]. In LUAD with the micropapillary pattern, CXCL14 expression in RNA level was identified as a possible diagnostic biomarker and associated with poorer prognosis of patients [27]. CXCL14 plays tumor-supportive or suppressive roles due to the differences in cell derivation. Smoking induced expression of CXCL14, which links chronic obstructive pulmonary disease to lung cancer [28]. Our study demonstrated that CXCL14 was upregulated in RNA (supplementary Fig. 1) and protein levels (Fig. 4, supplementary Fig. 2) in early-stage LUAD patients.

LUAD arises from multiple cells- of- origin. Many oncogenic drivers and co-occurring mutations, as well as the dysregulation of the microenvironment. Several genetic alterations (such as TP53, KRAS, KEAP1, STK11, ALK, and EGFR) were demonstrated to be involved in the initiation of LUAD. However, the mechanism of LUAD tumorigenesis has not yet been elucidated (30). Proteins that are secreted from the cell play a crucial role in many physiological, developmental, and pathological processes and are important for both intercellular and intracellular communication. The results of the present study showed that MDK, WFDC2, and CXCL14 were upregulated in early-stage LUAD and upregulated expressions of three secreted proteins promoted the proliferation of lung cancer cells, suggesting that these proteins might be involved in the tumorigenesis of LUAD.

In this study, we mainly focused on cancerogenic-related molecules that are easier to detect in biofluids. Down-regulated DEGs are also important .and their potential diagnostic values will be explored in the next research. In the current study, the number of plasma samples used for clinical validation is small, due to a limited number of patients with early-stage LUAD. Only three of 16 predicted proteins were demonstrated as upregulated proteins in the plasma of early-stage LUAD. The lack of statistical significance between the patients and healthy control might be partially related to the small size of clinical samples. Also, this study does not include other lung diseases (such as lung fibrosis, chronic obstructive pulmonary disease, etc.) as controls. The mechanism of expression regulation of these proteins in LUAD and whether they are regulated in other lung diseases remain unclear.

In summary, the present study demonstrated that MDK, WFDC2, and CXCL14 were upregulated in the plasma of early-stage LUAD and might be the candidate biomarkers for early diagnosis. The findings might be of great importance for the early diagnosis research of LUAD. The in vitro study of lung cancer cells indicated that upregulated expressions of the three proteins might play vital roles in tumorigenesis of early-stage LUAD, although more data and mechanism research are needed.

## Supplementary Information

**Additional file 1: Supplementary Fig. 1.** The mRNA expressions of the 16 selected secreted proteins in the TCGA dataset, including 515 LUAD patients (277 stage I, 125 stage II, 85 stage III, and 28 stage IV) and 59 normal controls. All the proteins were upregulated in the mRNA levels in the stage IA LUAD patients (*p* < 0.0001). **Supplementary Fig. 2. **The protein expressions of the 16 selected secreted proteins in the CPTAC dataset, including 111 LUAD patients (59 stage I, 30 stage II, 21 stage III, and 1 stage IV) and 11 normal controls. All the proteins were upregulated in stage I LUAD patients (*p* < 0.0001). **Supplementary Fig. 3.** Full-length gels of MDK, WFDC2, and CXCL14 expressions in A549 cells using GAPDH as an internal control.

## Data Availability

Data on plasma protein concentrations of the 26 stage I LUAD patients and 11 health donors are available from the authors upon reasonable request. All other data analyzed in this study were obtained from the GEO dataset GSE31210 and the UALCAN database (TCGA and CPTAC) as above-mentioned.

## References

[1] Sung H, Ferlay J, Siegel RL, Laversanne M, Soerjomataram I, Jemal A (2021). Global Cancer statistics 2020: GLOBOCAN estimates of incidence and mortality worldwide for 36 cancers in 185 countries. CA Cancer J Clin.

[2] Spella M, Stathopoulos GT (2021). Immune Resistance in Lung Adenocarcinoma. Cancers (Basel).

[3] Liang W, Chen Z, Li C, Liu J, Tao J, Liu X (2021). Accurate diagnosis of pulmonary nodules using a noninvasive DNA methylation test. J Clin Invest.

[4] Wang Y, Zhang L, Chen Y, Li M, Ha M, Li S (2020). Screening and identification of biomarkers associated with the diagnosis and prognosis of lung adenocarcinoma. J Clin Lab Anal.

[5] de Koning HJ, van der Aalst CM, de Jong PA, Scholten ET, Nackaerts K, Heuvelmans MA (2020). Reduced lung-Cancer mortality with volume CT screening in a randomized trial. N Engl J Med.

[6] Aberle DR, Adams AM, Berg CD, Black WC, Clapp JD, Fagerstrom RM (2011). Reduced lung-cancer mortality with low-dose computed tomographic screening. N Engl J Med.

[7] Ji G, Bao T, Li Z, Tang H, Liu D, Yang P (2021). Current lung cancer screening guidelines may miss high-risk population: a real-world study. BMC Cancer.

[8] Liu C, Xiang X, Han S, Lim HY, Li L, Zhang X (2022). Blood-based liquid biopsy: insights into early detection and clinical management of lung cancer. Cancer Lett.

[9] Su L, Zhao J, Su H, Wang Y, Huang W, Jiang X (2022). CircRNAs in lung adenocarcinoma: diagnosis and therapy. Curr Gene Ther.

[10] Yao Y, Zhang T, Qi L, Liu R, Liu G, Wang J (2020). Comprehensive analysis of prognostic biomarkers in lung adenocarcinoma based on aberrant lncRNA-miRNA-mRNA networks and cox regression models. Biosci Rep.

[11] Yao B, Qu S, Hu R, Gao W, Jin S, Liu M (2019). A panel of miRNAs derived from plasma extracellular vesicles as novel diagnostic biomarkers of lung adenocarcinoma. FEBS Open Bio.

[12] Cai Q, Zhang P, He B, Zhao Z, Zhang Y, Peng X (2020). Identification of diagnostic DNA methylation biomarkers specific for early-stage lung adenocarcinoma. Cancer Genet.

[13] Jin CY, Du L, Nuerlan AH, Wang XL, Yang YW, Guo R (2020). High expression of RRM2 as an independent predictive factor of poor prognosis in patients with lung adenocarcinoma. Aging (Albany NY).

[14] Zhang J, Zhang Y, Ma Z (2019). In silico prediction of human secretory proteins in plasma based on discrete firefly optimization and application to Cancer biomarkers identification. Front Genet.

[15] Fang R, Yang Y, Han H, Fu X, Dong L, Xie B (2018). Analysis of risk factors for stage I lung adenocarcinoma using low-dose high-resolution computed tomography. Oncol Lett.

[16] Kanehisa M, Goto S (2000). KEGG: Kyoto encyclopedia of genes and genomes. Nucleic Acids Res.

[17] I H, Cho JY. (2015). Lung Cancer biomarkers. Adv Clin Chem.

[18] Zhang ZZ, Wang G, Yin SH, Yu XH (2021). Midkine: a multifaceted driver of atherosclerosis. Clin Chim Acta.

[19] Filippou PS, Karagiannis GS, Constantinidou A (2020). Midkine (MDK) growth factor: a key player in cancer progression and a promising therapeutic target. Oncogene..

[20] Yuan K, Chen Z, Li W, Gao CE, Li G, Guo G (2015). MDK protein overexpression correlates with the malignant status and prognosis of non-small cell lung Cancer. Arch Med Res.

[21] Wang Z, Li Z, Zhou K, Wang C, Jiang L, Zhang L (2021). Deciphering cell lineage specification of human lung adenocarcinoma with single-cell RNA sequencing. Nat Commun.

[22] Liu Q, Liu DW, Zheng MJ, Deng L, Wang HM, Jin S (2021). Human epididymis protein 4 promotes Pglycoproteinmediated chemoresistance in ovarian cancer cells through interactions with Annexin II. Mol Med Rep.

[23] Galgano MT, Hampton GM, Frierson HF (2006). Comprehensive analysis of HE4 expression in normal and malignant human tissues. Mod Pathol.

[24] Zhang T, Long H, Li J, Chen Z, Wang F, Jiang SW (2020). WFDC2 gene deletion in mouse led to severe dyspnea and type-I alveolar cell apoptosis. Biochem Biophys Res Commun.

[25] Lv M, Wang F, Wang X, Zhang C (2019). Diagnostic value of human epididymis protein 4 in malignant pleural effusion in lung cancer. Cancer Biomark.

[26] Gowhari SA, Haleem A-QZ, Markov A, Valerievich YA, Ezzatifar F, Ahmadi M (2021). Chemokine CXCL14; a double-edged sword in cancer development. Int Immunopharmacol.

[27] Sata Y, Nakajima T, Fukuyo M, Matsusaka K, Hata A, Morimoto J (2020). High expression of CXCL14 is a biomarker of lung adenocarcinoma with micropapillary pattern. Cancer Sci.

[28] Seguin L, Durandy M, Feral CC (2022). Lung adenocarcinoma tumor origin: a guide for personalized medicine. Cancers (Basel)..

