# Supplementary material for: Secreted proteins MDK, WFDC2, and CXCL14 as candidate biomarkers for early diagnosis of lung adenocarcinoma
Source: BMC Cancer. 2023 Jan 31;23:110. doi: 10.1186/s12885-023-10523-z (PMC9887767; doi:10.1186/s12885-023-10523-z)
Supplement: Supplementary file 1 — Additional file 1: Supplementary Fig. 1. The mRNA expressions of the 16 selected secreted proteins in the TCGA dataset, including 515 LUAD patients (277 stage I, 125 stage II, 85 stage III, and 28 stage IV) and 59 normal controls. All the proteins were upregulated in the mRNA levels in the stage IA LUAD patients (p < 0.0001). Supplementary Fig. 2. The protein expressions of the 16 selected secreted proteins in the CPTAC dataset, including 111 LUAD patients (59 stage I, 30 stage II, 21 stage III, and 1 stage IV) and 11 normal controls. All the proteins were upregulated in stage I LUAD patients (p < 0.0001). Supplementary Fig. 3. Full-length gels of MDK, WFDC2, and CXCL14 expressions in A549 cells using GAPDH as an internal control. [file 12885_2023_10523_MOESM1_ESM.docx]

**Supplementary materials**

**Supplement Figure 1 The mRNA expressions of the 16 selected secreted proteins in the TCGA dataset, including** **515 LUAD patients (277 stage I, 125 stage II, 85 stage III, and 28 stage IV) and 59 normal controls.** All the proteins were upregulated in the mRNA levels in the stage IA LUAD patients (p<0.0001).

**Supplement Figure 2 The protein expressions of the 16 selected secreted proteins in the CPTAC dataset, including 111 LUAD patients (59 stage I, 30 stage II, 21 stage III, and 1 stage IV) and 11 normal controls.** All the proteins were upregulated in the stage I LUAD patients (p<0.0001).

**Supplementary Figure 3** Full-length gels of MDK, WFDC2 and CXCL14 expressions in A549 cells.
